# Supplementary material for: Evaluation of the association between postural control and sagittal curvature of the spine
Source: PLoS One. 2020 Oct 27;15(10):e0241228. doi: 10.1371/journal.pone.0241228 (PMC7591056; doi:10.1371/journal.pone.0241228)
Supplement: Attachment — Submitted filename: Response to Reviewers.docx [file pone.0241228.s003.docx]

**Dear Editor and dear Reviewers,**

Thank you for your thorough assessment and valuable tips on the complex work, entitled: "Efficiency of Compensation Reactions in Children by the Extent of Thoracic Kyphosis and Lumbar Lordosis ".

We are of the opinion that all suggestions given are correct and their application will positively influence the comprehensibility of the text and clarity of the message. Therefore, during changes in the text, we tried to apply as closely as possible all the comments provided.

In the remainder of the letter I will try to answer in detail how the individual suggestions were responded, in the order in which they were submitted.

**Answers to Reviewer #1**

The authors undertook a comprehensive review of their article and addressed my comments in a satisfactory way. Their manuscript is now better structured and easier to read. It contains valuable information and therefore I recommend this manuscript for publication.
However, there still remains a minor critical comment. I would still suggest omitting the following phrases as they stray away from the main theme and contain controversial statements:
Page 16: Body posture disorders can affect various systems of the human body [52], from the
pain accompanying postural dysfunctions [53-56], through pulmonary ventilation disorders
[57] and intestinal motility disorders, to malocclusion [54]. The relationship between spinal
curvature and balance disorders has been confirmed in numerous studies [29, 30, 54, 58, 59].
Page 17: Most authors use the Romberg test to assess body balance. However, the ability to use vision to improve motor skills in children during development is only emerging and both values can be aligned closely [61].

Answer: Thank you for this suggestion. As suggested, this part has been removed.

**Answers for Reviewer 2.**

Thank you for revising the manuscript and incorporating the suggested revisions. The following revisions are further recommended:

1. Title: Revise the title, as it is not clear. It is suggested that the revised title more closely reflect the analysis. For example' Evaluation of the association between postural control and sagittal curvature of the spine '.

Answer: Thanks for the suggestion. The title has been changed as proposed.

2. Abstract: revise the text:

‘worse’ with ‘smaller’

‘coronary’ with coronal

Answer: Thanks for the suggestion. Words changed: 'worse' with 'greater', 'coronary' with coronal '. Worse in this case meant a longer diplacement in all directions, so instead of 'worse' we put 'greater'.

3. Introduction:

1. Revise: ‘ The physiological values…’ to ‘ The amplitude of ‘

Thanks for the suggestion. The text has been changed as proposed.

2. Combine 2nd and 3rd paragraphs. ‘ Body posture disorders…..’ with the next paragraph starting with ‘The literature’….

Answer: Thanks for the suggestion. The text has been changed as proposed.

3. It is important to better distinguish the displacement of the COP under both feet during upright quiet standing, with that of the COP displacement under 1 foot during walking. Although this is described in paragraph 4, it is recommended to revise this paragraph to be more clear.

For example:

1. COP displacement during quiet standing reflects the net contribution of the CNS to control the movement of the Centre of Mass. Where the horizontal acceleration of the COM is proportional to the difference between the COP-COM. (Winter et al., ) during quiet stance it is the hip abductors/adductors that generate torque to control ML movement, and it is the ankle plantar flexors/dorsiflexors that control A/P movement

2. During walking, the movement of the COP moves from posterior to anterior under the foot, and ML. Although the above concept holds true (ie., COP reflects net output of CNS control), how it does this is more complex given the multiple degrees of freedom, and joint motions.

In light of this, it is suggested to revise this paragraph to further strengthen the rational as to why the investigators chose the COP as the primary outcome variable to measure standing stability, and also stability during walking.

Answer: Thank you for this suggestion. Section 4 has been supplemented with the proposed content.

Methods:

1. Clarify for how long the centre of pressure was recorded. Was it 3 seconds, the same as the rastersterography, or a different length of time.

Answer: Thank you for this suggestion. the text was supplemented with the sentence 'The measurement was carried out for 3 seconds at the same time as the shape of spine was assessed with the DIERS formetric system.'

2. Research methodology: It is recommended to define each parameter that was included in the analysis. For example, Table 2 contains variables such as ‘Maximum displacement of centre of gravity in coronal plane in pressure gait’. This is very difficult to understand what this variable is in the table. Therefore, each measure described in Table 2 should be defined in the methods.

a. Angle of Thoracic Kyphsosi, Angle of lumbar Lordosis

b. Displacement of centre of gravity in coronal/sagittal planes in pressure gait

c. Maximum displacement of centre of pressure to side [mm].

Answer: Thank you for this valuable suggestion. The parameters were described and their nomenclature arranged throughout the manuscript.

Results:

1. Table 2, 3 and 4 are redundant. It is recommended to include primarily Table 4.

Answer: Thank you for the suggestion. Tables removed as recommended.

2. Revise the variable names to be consistent with the definition in the methods, and also how COP displacement is referred to throughout the manuscript. For example:

a. ‘Maximum displacement of center of gravity in coronal plane in pressure gait’. Suggest revising to ‘Maximum displacement of centre of pressure in coronal plane during gait’.

b. ‘Maximum displacement of center of gravity in sagittal plane in pressure gait’. Suggest revising to ‘Maximum displacement of centre of pressure in sagittal plane during gait’.

Answer: Thank you for these suggestions. The text was corrected as recommended.

Discussion

- It is suggested to use consistent terminology with respect to COP displacement. Rather than using terms such as ‘swing’, ‘deflections’, it is suggested to consistently the word ‘displacement’ throughout the text.

Thank you for these suggestions. The text was corrected as recommended.

- Revise – Nalut, and Naulut to correct reference ‘ Nault’.

Answer: Thank you for these suggestions. The text was corrected as recommended.

- Revise statement ‘ Rougier et al., explains this phenomenon by the fact that swing during walking is changed by the forces tramsitted to the feet and are partly reduced by compensations occurring in the hip and ankle’. It is not clear what ‘swing’ means. Is this the swing phase of gait?

Answer: Thank you for these suggestions. The text was corrected as recommended. The statement 'Rougier et al., Explains this phenomenon by the fact that swing during walking is changed by the forces tramsitted to the feet and are partly reduced by compensations occurring in the hip and ankle ’changed to' Rougier et al. this phenomenon by the fact that CoG displacement during walking is changed by the forces transmitted to explain the feet and are partly reduced by compensations occurring in the hip and ankle '.

Thank you for the opportunity to make changes in our manuscript. We have referred to all your comments and suggestions. We believe, that in current form, our manuscript meets the standards of PLOS ONE.

Sincerely,

the Authors.
